# Factors underlying Dutch farmers' intentions to adapt their agronomic management to reduce *Fusarium* species infection in wheat

**DOI:** 10.1371/journal.pone.0237460

**Published:** 2020-09-10

**Authors:** E. M. Janssen, M. C. M. Mourits, H. J. van der Fels-Klerx, A. G. J. M. Oude Lansink

**Affiliations:** 1 Business Economics Group, Wageningen University & Research, Wageningen, the Netherlands; 2 Wageningen Food Safety Research (WFSR), Wageningen University & Research, Wageningen, the Netherlands; Universita degli Studi di Pisa, ITALY

## Abstract

Infection of wheat by *Fusarium* species can lead to Fusarium Head Blight (FHB) and mycotoxin contamination, thereby reducing food quality and food safety, and leading to economic losses. Agronomic management through the implementation of various pre-harvest measures can reduce the probability of *Fusarium* spp. infection in the wheat field. To design interventions that could stimulate wheat farmers to (further) improve their agronomic management to reduce FHB, it is key to understand farmers’ behaviour towards adapting their management. The aim of this paper was to understand the intention, underlying behavioural constructs, and beliefs of Dutch wheat farmers to adapt their agronomic management to reduce FHB and mycotoxin contamination in wheat, applying the Theory of Planned Behaviour (TPB). Data were collected from 100 Dutch wheat farmers via a questionnaire. The standard TPB analysis was extended with an assessment of the robustness of the belief results to account for the statistical validity of the analysis on TPB beliefs (i.e. to address the so-called expectancy-value muddle). Forty-six percent of the farmers had a positive intention to change their management in the next 5 years. The two behavioural constructs significantly related to this intention were attitude and social norm, whereas association with the perceived behavioural control construct was insignificant indicating that farmers did not perceive any barriers to change their behaviour. Relevant attitudinal beliefs indicated specific attributes of wheat, namely yield, quality and safety (lower mycotoxin contamination). This indicates that strengthening these beliefs—by demonstrating that a change in management will result in a higher yield and quality and lower mycotoxin levels—will result in a stronger attitude and, subsequently, a higher intention to change management. Interventions to strengthen these beliefs should preferably go by the most important referents for social norms, which were the buyers and the farmer cooperatives in this study.

## Introduction

In the Netherlands, wheat is cultivated on around 7,000 farms with an annual total size of 120,000 ha [1]. During wheat cultivation, *Fusarium* species can infect the grain, which can lead to Fusarium Head Blight (FHB), decreasing the yield and quality of the wheat produced. Moreover, *Fusarium* spp. can produce mycotoxins, like deoxynivalenol (DON), zearalenone and nivalenol, which are fungal toxic secondary metabolites that can cause adverse health effects in animals and humans upon consumption of the grain [2]. Mycotoxin contamination of wheat is regularly reported in the Netherlands [3–6] and other countries [7]. Hence, legal maximum limits for the presence of mycotoxins in feed and food have been set in Europe and other parts of the world to protect animal and human health [8]. In spite of these limits, human exposure assessments in Europe have shown that the intake of some mycotoxins is currently above the tolerable daily intake for certain vulnerable groups [7, 9]. The past four years, two large European Union's Horizon 2020 funded research and innovation projects focussed on further reducing mycotoxin contamination in feed and food commodities like wheat (www.mycokey.eu and www.mytoolbox.eu).

Given the difficulty in removing mycotoxins during subsequent processing downstream the wheat supply chain, mycotoxin management is mainly focused on agronomic management at the arable farm [10]. *Fusarium* spp. present on plant debris can survive and contaminate the next planted crop. Various pre-harvest measures can be taken to reduce the probability of *Fusarium* spp. infection and related mycotoxin contamination, like decontamination of seeds, crop rotation, soil cultivation, selection of a wheat variety resistant to *Fusarium* spp. infection, and fungicide use [2, 11, 12]. Decontaminated seeds are used to avoid initial fungal contamination by infected seeds [13]. Tillage and ploughing bring the contaminated plant debris deeper into the soil which can avoid contamination of the next planted crop. In addition, a crop rotation scheme in which *Fusarium* spp. infection prone crops are not succeeding each other will decrease the chance of recontamination as well. The most effective fungicide application time was reported to be around the wheat flowering stage [14–16]. Additional pre-harvest measures, like novel biological control measures are currently under development and being tested, see reviews of Shah *et al*. [17] and Torres *et al*. [18]. An effective way to prevent and control *Fusarium* spp. infection and mycotoxin contamination in wheat is to use an integrated approach consisting of a combination of effective pre-harvest measures, like the combination of fungicide use during flowering, selection of a *Fusarium* resistant variety, and ploughing or crop rotation [10, 19–24].

Current implementation of pre-harvest measures to reduce FHB and mycotoxin contamination in wheat by Dutch farmers was studied by Janssen *et al*. [25]. This study showed that most Dutch farmers applied more than six pre-harvest measures against *Fusarium* spp. infection, while only half of the farmers used an effective integrated approach. This study also showed that 79% of the farmers used fungicides throughout the whole cultivation period whereas only 6% of the farmers used fungicides only during the flowering stage. In addition, 20% of the farmers used biological control measures [25].

The optimal mycotoxin management might not fit ‘sustainable agriculture’ [26] in which, for example, conservation tillage is in contrast to the effective mycotoxin reduction approach of (deep) ploughing to burry soil debris to reduce *Fusarium* spp. infection and the use of fungicides throughout the whole cultivation period. Overall, it is expected that farmers will adapt their agronomic management in the future, for instance, to comply to new regulations, to accommodate environmental policies and/or to become more (cost-)effective. An adaptation in agronomic management to reduce FHB and mycotoxin contamination can entail the future implementation of less, more or different pre-harvest measures. These adaptations could be directed to a more effective integrated approach or a reduction in fungicide use by concentrating its use around the sensitive flowering stage or by adopting biological measures.

To be able to design interventions that adapt agronomic management for *Fusarium* spp. infection reduction, it is key to understand farmers’ behaviour towards agronomic management. A frequently deployed method for understanding farmers’ behaviour is the Theory of Planned Behaviour (TPB) with underlying behavioural constructs following from personal beliefs [27]. Knowledge of the underlying constructs and beliefs provides information on how to stimulate this behaviour and can provide targets for setting up incentive mechanisms. Several behavioural studies used the TPB to investigate the intentions, behavioural constructs and beliefs of farmers to manage, for example, grassland [28], pathogen invasions in horticulture [29], diseases in animals [30–32] or agri-environmental measures [33]. Although the studies mentioned above give insight into farmers behaviour regarding agronomical measures, sector- and farm-specific differences were found [28, 29, 33, 34]. This makes information on how to stimulate a certain behaviour unique to the target group and the farm and agricultural sector at hand. To date, similar studies have not been performed on agronomic management to reduce FHB and mycotoxins in wheat or other cereal crops. This study aimed to explore the intention and underlying behavioural constructs and beliefs of Dutch wheat farmers to adapt their future agronomic management to reduce FHB and mycotoxins in wheat. A questionnaire was developed based on the TPB and this questionnaire was distributed among a sample of Dutch arable farmers. The relation between the underlying behavioural constructs and intention was analysed, followed by an analysis of the relations between the beliefs and underlying constructs. This latter analysis was extended with an assessment of the robustness of the belief results, because the statistical validity of the standard analysis of the TPB beliefs has been questioned, i.e. the so-called expectancy-value muddle [34–37].

## Methods

### Theory of Planned Behaviour (TPB)

**Fig 1:**
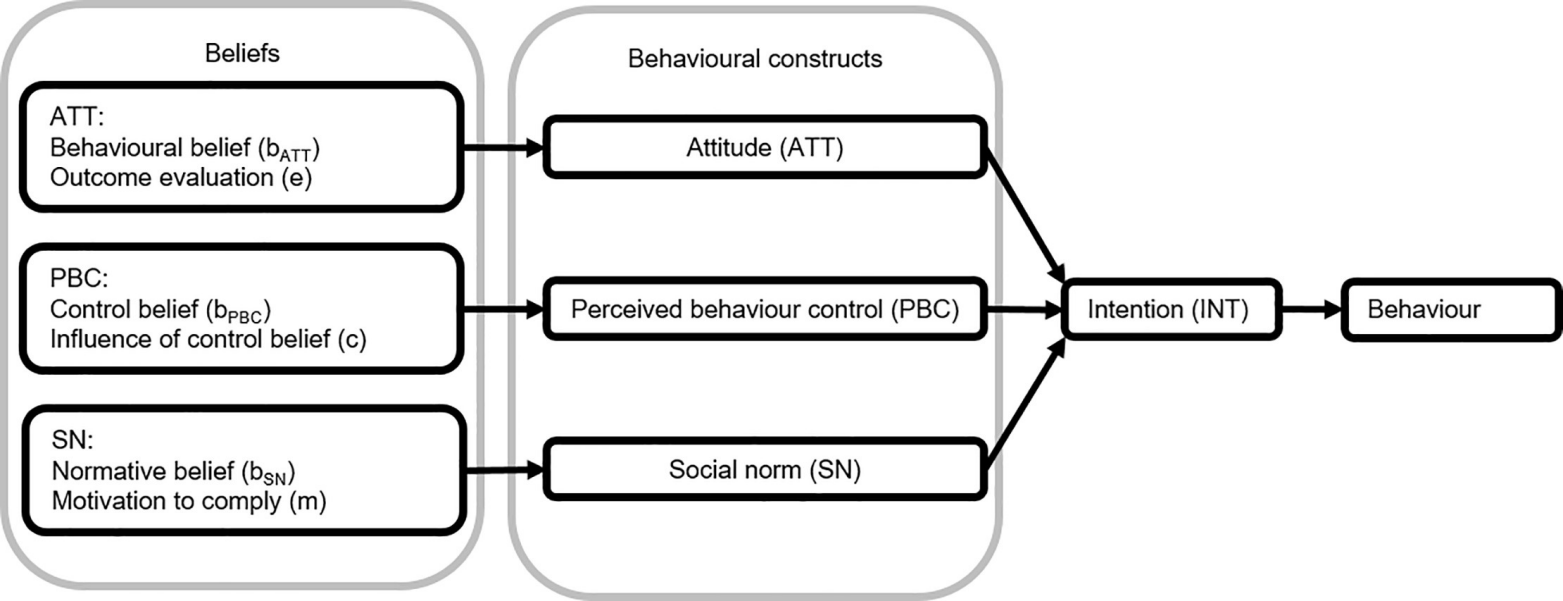
Theory of planned behaviour [27, 38].

Intentions are a proximal measure of future behaviour. The stronger the intention is, the more likely the behaviour will be executed in the future. According to the TPB, intentions (INT) can be determined by three behavioural constructs: (i) attitude (ATT), (ii) perceived behavioural control (PBC), and (iii) subjective norm (SN) (Fig 1). ATT provides insight into the positive or negative attitudes towards the behaviour. PBC accounts for factors outside one’s control that affect behaviour, and SN accounts for the perceived social pressure. The stronger these behavioural constructs, the stronger an individual’s intention to perform the behaviour.

Each of the behavioural constructs follows from personal beliefs (Fig 1); beliefs about the likely consequences of the behaviour (ATT beliefs), beliefs about the factors that may stimulate or prevent performance of the behaviour (PBC beliefs), and beliefs about the expectations of others (SN beliefs) [39]. These beliefs help to understand the drivers of farmers’ behaviour and provide targets for intervention [40]. Targeting ATT beliefs can change a person’s attitude towards the behaviour in question, and PBC beliefs provide insight into the actual barriers to perform the behaviour. SN beliefs will indicate the most important people (termed referents) in a person’s decision to adapt his/her behaviour, which is useful in understanding the channels that are suited for implementing incentive mechanisms.

Overall, the TPB is a well-known method to gain insight into behaviour and allows for quantitative research by use of data obtained by questionnaires [27, 41]. Knowledge of the underlying constructs and beliefs provides information on how to stimulate this behaviour and can provide targets for incentive mechanisms.

### Survey

Data on TPB variables, INT, ATT, PBC and SN, and underlying beliefs were collected from 100 Dutch wheat farmers using an online questionnaire in Dutch in 2017. The mean arable land of these farmers was 95.3 ha ranging from 17.5 to 230 ha. Wheat was the main crop for 15% of the farmers, 26% produced wheat for food, and 67% experienced a *Fusarium* spp. infection in the past five years [25]. The questionnaire was developed based on the TPB [38] with questions on INT, ATT, PBC and SN, and questions on belief statements. These belief statements were elicitated from commonly held beliefs during ten qualitative face-to-face interviews with farmers and added to the questionnaire. The focus of the questionnaire was on the intention of the following farmers’ behaviour: ‘to adapt the approach to reduce *Fusarium* spp. infection in the coming 5 years’. The specific questions and answer formats related to the TPB variables in the questionnaire are presented in Tables 1 and 2. INT, ATT, PBC and SN were measured on a 5-point textual bipolar Likert scale (e.g. strongly disagree–somewhat disagree–neither agree nor disagree–somewhat agree–strongly agree). Each belief statement related to ATT, PCB and SN was measured by a belief score (b) and an outcome score; i.e. outcome evaluations (e) for ATT beliefs, the influence of control beliefs (c) for PBC beliefs, and the motivation to comply (m) for SN beliefs. Each answer on the belief statement was measured with a 5-point textual Likert answer scale representing either a unipolar scale (e.g. unlikely–slightly likely–somewhat likely–very likely–extremely likely) or a bipolar scale (e.g. very unimportant–moderately unimportant–neutral–moderately important–very important) (Table 2).

**Table 1:** Theory of Planned Behaviour (TPB) questionnaire questions and answer formats for intention (INT) and behavioural constructs attitude (ATT), perceived behavioural control (PBC), and social norm (SN).

TPB	Question	Possible answer– 5-point scale^a^
**INT**	I expect to change my approach to reduce *Fusarium* infection in the coming 5 years.	Strongly disagree <> Strongly agree
	I plan to change my approach to reduce *Fusarium* infection in the coming 5 years.	Strongly disagree <> Strongly agree
	I want to change my approach to reduce *Fusarium* infection in the coming 5 years.	Strongly disagree <> Strongly agree
**ATT**	Changing my approach to reduce *Fusarium* infection is…	Harmful <> Beneficial
	Changing my approach to reduce *Fusarium* infection is…	Necessary <> Unnecessary
	Changing my approach to reduce *Fusarium* infection is…	Unimportant <> Important
**PBC**	I have enough possibilities to change my approach to reduce *Fusarium* infection.	Strongly disagree <> Strongly agree
	I can change my approach to reduce *Fusarium* infection if I want to do so.	Strongly disagree <> Strongly agree
	Changing my approach to reduce *Fusarium* infection is up to me and not dependent on other aspects.	Strongly disagree <> Strongly agree
**SN**	Persons that are dealing with me expect that I change my approach to reduce *Fusarium* infection.	Strongly disagree <> Strongly agree
	I feel social pressure to change my approach to reduce *Fusarium* infection.	Strongly disagree <> Strongly agree
	Persons who are important to me, think that I should change my approach to reduce *Fusarium* infection.	Strongly disagree <> Strongly agree

^a^The answer formats were text only and reflected a bipolar answer scale (<>).

**Table 2:** Theory of planned behaviour (TPB) beliefs questionnaire questions and answer formats for attitude (ATT) beliefs, perceived behavioural control (PBC) beliefs, and social norm (SN) beliefs.

Beliefs	Question	Possible answer– 5-point scale^a^
ATT: behavioural beliefs (b_ATT_)	Changing my approach to reduce *Fusarium* infection. . .	
	…results in a higher wheat quality	Unlikely >> extremely likely
	…results in a higher wheat yield	Unlikely >> extremely likely
	…results in lower mycotoxin (DON) contamination in wheat	Unlikely >> extremely likely
	…is not cost-effective	Unlikely >> extremely likely
	…is pointless because of the unpredictability of the weather	Unlikely >> extremely likely
**ATT: outcome evaluation (e)**	Will the <*attitudinal statement; e*.*g*. *higher wheat quality*, *higher wheat yield*, *etc*.> be important in your decision to change your approach to reduce *Fusarium* spp. infection?	Very unimportant <> Very important
PBC: control beliefs (b_PBC_)	In order to change my approach. . .	
	. . .enough alternative preventive measures are available	Unlikely >> extremely likely
	. . .enough cost-effective methods are available	Unlikely >> extremely likely
	. . .I have sufficient knowledge	Unlikely >> extremely likely
	. . .I have enough possibilities to obtain individual advice	Unlikely >> extremely likely
	. . .I have sufficient equipment and manpower	Unlikely >> extremely likely
	. . .I have sufficient financial scope to invest	Unlikely >> extremely likely
**PBC: influence of control belief (c)**	Do <*PBC statements; e*.*g*. *‘the availability of enough alternative preventive measures’*, *‘having sufficient knowledge’*, *etc*.> make it more difficult (prevent you) or easier for you (persuade you) to change your approach?	Extremely more difficult (prevent) <> Extremely easier (enable me)
SN: normative beliefs (b_SN_)	What are the opinions of the following social referents (e.g. fellow farmers) about you changing your approach to reduce *Fusarium* infection?	Strongly oppose <> Strongly favour OR not applicable
	• Fellow farmers	
	• Members of your study group	
	• Your buyer	
	• Farmer cooperative	
	• Independent advisor	
	• Government official	
	• RVO^b^	
	• Fungicide supplier	
	• Scientists	
	• Environmental organisation	
	• Consumer	
	• Family members/friends	
**SN: motivation to comply (m)**	Is the opinion of < social referent, e.g. fellow farmers> important in your decision to change your approach to reduce *Fusarium* infection?	Unimportant >> Extremely important

^a^The answer formats were text only and reflected a bipolar answer scale (<>) or a unipolar answer scale (>>).

^b^Rijksdienst voor Ondernemend Nederland (Netherlands Enterprise Agency) is part of the Ministry of Economic Affairs and Climate Policy and works at the instigation of ministries and the European Union, aiming to improve opportunities for entrepreneurs and to strengthen their position [42].

Questions were part of a more extensive questionnaire that covered related research topics, like farm(er) characteristics and implementation of pre-harvest measures [25], (cost-) effectiveness of pre-harvest measures, and incentive mechanisms among European wheat farmers as part of the European Union's Horizon 2020 MyToolbox project [43]. The questionnaire was pre-tested by three Dutch farmers for clarity and consistency. Their feedback was used to adapt the online questionnaire. The link to the online questionnaire was distributed via farmers’ associations by email and newsletters to Dutch wheat farmers. Farmers were incentivised to participate in the survey by offering them the chance of winning one of ten €25,—gift vouchers. Farmers could give their email address voluntarily for future contact, and all personal information was stored separately from the questionnaire output. The study protocol and consent procedure complied with the Netherlands Code of Conduct for Scientific Practice. It was approved by the Social Sciences Ethics Committee of the Wageningen University (CoC number 09131098).

### Data analysis

#### Intention and behavioural constructs

INT and the behavioural constructs ATT, PBC, and SN were each composed of a series of three similar questions (Table 1) measured on a bipolar textual answer scale which was converted to a numeric score ranging from -2 to 2. For INT, ATT, PBC, and SN, each of their respective answer scores (based on three questions each, see Table 1) were measured by Cronbach alpha [44] to confirm that they were internally consistent (Cα> 0.7) and then combined into a single composite score by averaging the scores.

A common approach to study TPB results to understand the relationship between the different behavioural constructs and the intention (INT) of farmers is the use of a linear regression model [41]. A censored linear regression model was performed with INT as the dependent variable (which is a left and right censored variable (respectively at -2 and 2)), and ATT, PBC, and SN as the explanatory variables:

$$y\;(INT)=\beta_{0}+\beta_{1}\times ATT+\beta_{2}\times PBC+\beta_{3}\times SN$$ (1)

In addition to the regression analysis, the percentage of farmers with a negative, neutral, or positive view towards INT, ATT, PBC, and SN were determined. A score below 0 was labelled ‘negative’, a score equal to 0 was ‘neutral’, and a score above 0 was ‘positive’.

#### Beliefs

Beliefs were measured by two composites: (i) the strength of a belief score (b_ATT_, b_PBC_, or b_SN_) and (ii) an outcome score (e, c, or m) (Table 2). It is common to multiply the belief score (expectancy) with the outcome score [27]—also called the expectancy-value model—to measure a belief, resulting in a multiplicative composite:

$$ATT\;belief=b_{ATT}\times e$$ (2)

$$PBC\;belief=b_{PBC}\times c$$ (3)

$$SN\;belief=b_{SN}\times m$$ (4)

This multiplicative composite of the belief can be used in a regression or correlation analysis to find the beliefs significantly related to their behavioural construct. However, several studies questioned the statistical validity of the expectancy-value model’s multiplicative composite, the so-called expectancy-value muddle [45–48]. For example, the size of correlation coefficients between a belief and its construct can vary according to the answer scale, especially when the scale includes zero as in a bipolar answer scale. Also, beliefs calculated as multiplicative composites are difficult to interpret and do not represent what is intended by the TPB [41]. A suggested solution is to avoid the use of the multiplicative composites and only use the basic belief score (b) [46–48].

So, literature is not conclusive on which answer scale to use to calculate the multiplicative composites. Hence, in this study, results from the basic belief score and several multiplicative composites based on different answer scales were compared to the results from the multiplicative composite based on the textual scale used in the questionnaire (Table 2), to check the robustness of the results. Therefore, the textual belief- and outcome scores were converted to both a unipolar (1 to 5) and a bipolar answer scale (-2 to 2), independent of their textual answer scale. Thus, four different multiplicative composites of the beliefs were calculated: (i) unipolar belief score × unipolar outcome score (uni-uni); (ii) unipolar belief score × bipolar outcome score (uni-bi); (iii) bipolar belief score × unipolar outcome score (bi-uni); (iv) bipolar belief score × bipolar outcome score (bi-bi). Correlation coefficients were calculated to understand which beliefs underlie the behavioural constructs of Dutch wheat farmers. Per belief, a correlation coefficient was calculated between the belief score (b) and their corresponding behavioural construct (ATT, PBC or SN), and between the four multiplicative composites and their corresponding construct.

#### Salient beliefs

In addition to knowing which beliefs underlie the behavioural constructs, the number of farmers with a salient individual belief was determined. If a belief is salient, it means that farmers already hold this belief and that strengthening this belief will result in a stronger behavioural construct. The selection of salient beliefs could be determined by individual respondents or by elicitation of commonly held beliefs among a sample group [49]. Commonly held beliefs from the elicitation study were classified as salient based on the score of individual farmers for the outcome scores. For ATT beliefs, those farmers were selected who think that the belief is important in their decision, i.e. if the score of outcome evaluation (e) > 0 when measured on a -2 to 2 scale. Farmers had salient PBC beliefs if they indicated that the belief makes the decision either easier or more difficult, i.e. the influence control (c) ≠ 0 on a -2 to 2 scale. Farmers had a salient SN belief if they indicated not applicable (NA) at the belief score (b) question or selected in the motivation to comply question (m) that a referent is important in their decision (m > 1 on a 1 to 5 scale).

## Results

### Intention

**Fig 2:**
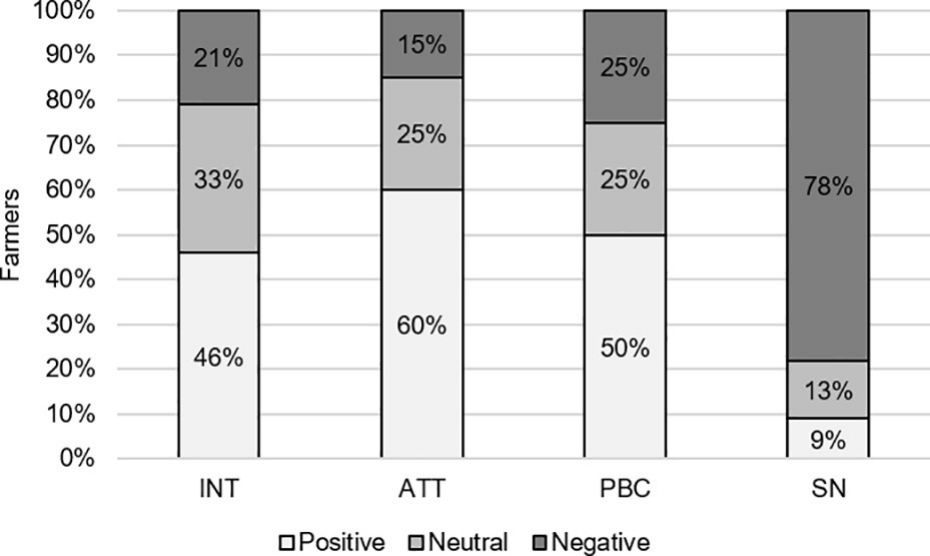
Intention (INT) of farmers towards adapting their *Fusarium* spp. management, and their underlying attitude (ATT), perceived behavioural control (PBC), and social norm (SN) (% negative, neutral, or positive).

Most Dutch farmers had a positive (46%) or a neutral (33%) intention to adapt their agronomic management to reduce FHB and mycotoxin contamination in the coming five years (INT), while 21% of the farmers had a negative intention towards a future adaptation (Fig 2). Sixty percent of the farmers had a positive ATT towards adapting their agronomic management, while fifteen percent had a negative ATT. About 50% of the farmers scored positive for PBC. Another 25% scored negative for PBC. Most farmers (78%) did not feel social pressure to adapt; Fig 2 shows that only 9% scored positively for SN.

### Explanation of intention by behavioural constructs

**Fig 3:**
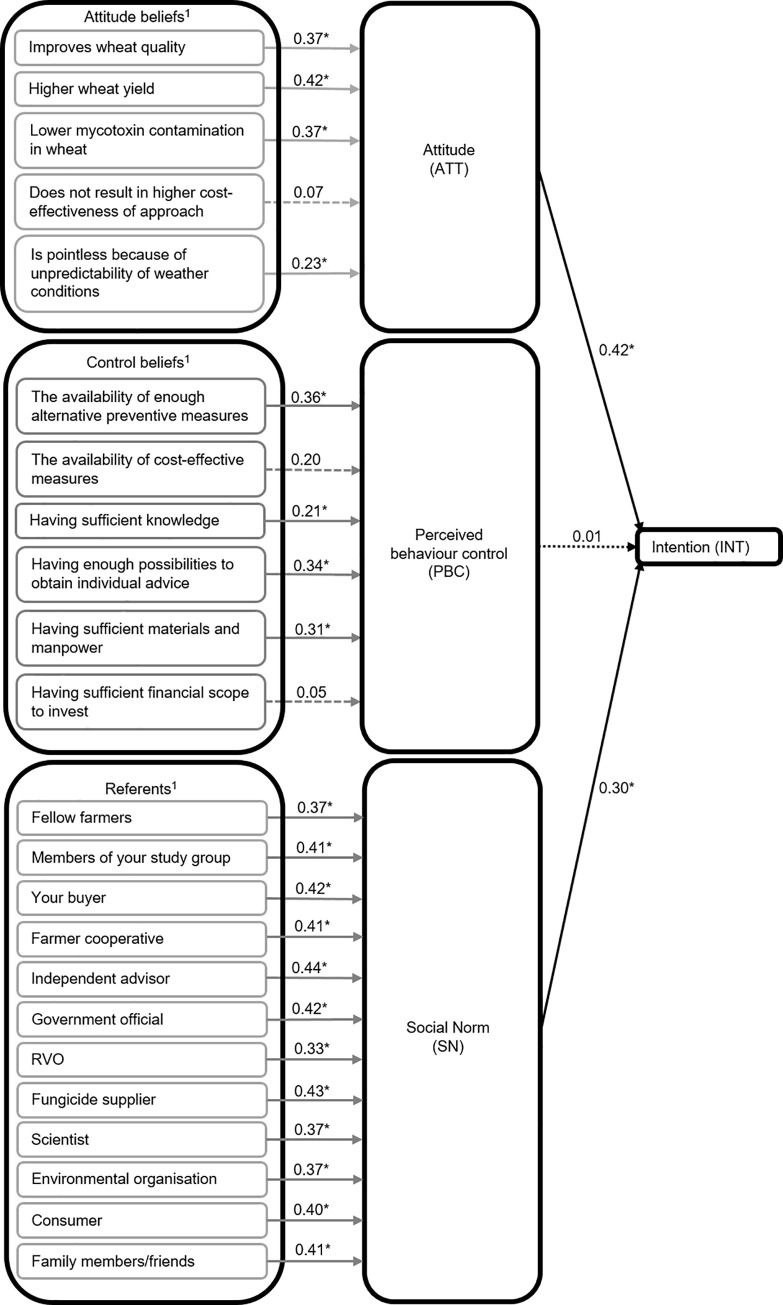
Regression and correlation coefficients of the TPB analysis. Regression coefficients (black arrows) of attitude (ATT), perceived behaviour control (PBC), and social norm (SN) as independent variables and intention as the dependent variable. Correlation coefficients (grey arrows) between the ATT, PCB and SN beliefs and their respective behavioural construct. *p<0.05; ^1^Multiplicative composite of the beliefs reflecting the questionnaire answer scale: uni-bi = unipolar belief score × bipolar outcome score for ATT and PBC beliefs and bi-uni = bipolar belief score × bipolar outcome score for SN referents.

The results of the censored regression of ATT, PBC, and SN on intention are shown in Fig 3. ATT was related to the intention (INT) with a positive regression coefficient of 0.42. SN was related to the intention with a positive coefficient of 0.30, and PBC was not significantly related to intention.

### Beliefs

The ATT beliefs with the highest scores were ‘higher wheat yield’ and ‘lower mycotoxin contamination in wheat’ (Table 3). The belief that was salient for most farmers (87%) was ‘higher wheat yield’. The PBC beliefs with the highest scores were ‘having enough possibilities to obtain individual advice’ and ‘having sufficient materials and manpower’ (Table 4). The PBC beliefs salient for most farmers were ‘the availability of enough alternative preventive measures’ (78%) and ‘the availability of cost-effective measures’ (76%). The referents salient for most farmers were buyers and farmer cooperative search with 87%, followed by the consumer with 74% of the farmers (Table 5).

**Table 3:** Average score for attitudinal (ATT) beliefs and the percentage of farmers for which the belief is salient (n = 92).

	Behavioural beliefs (b) Answer scale [1 to 5]		Outcome evaluations (e) Answer scale [-2 to 2]		Salient
	Mean	Stdev	Mean	Stdev	% of farmers
**Higher wheat quality**	3.2	1.1	1.0	0.8	77
**Higher wheat yield**	3.3	1.2	1.3	0.8	87
**Lower mycotoxin contamination in wheat**	3.5	1.1	1.0	0.8	78
**Does not result in higher cost-effectiveness of approach**	2.8	1.1	1.1	0.9	78
**Is pointless because of unpredictability of weather conditions**	3.0	1.2	0.9	0.9	65

**Table 4:** Average score for perceived behavioural control (PBC) beliefs and the percentage of farmers for which the belief is salient (n = 85).

	Control beliefs (b) Answer scale [1 to 5]		Influence of control beliefs (c) Answer scale [-2 to 2]		Salient
	Mean	Stdev	Mean	Stdev	% of farmers
**The availability of enough alternative preventive measures**	2.4	1.0	0.7	1.0	78
**The availability of cost-effective measures**	2.2	0.9	0.7	1.1	76
**Having sufficient knowledge**	2.8	1.1	0.5	0.9	69
**Having enough possibilities to obtain individual advice**	3.5	1.0	0.6	0.8	58
**Having sufficient materials and manpower**	3.5	1.0	0.5	0.7	53
**Having sufficient financial scope to invest**	3.1	1.1	0.6	0.9	57

**Table 5:** Average score social norm (SN) beliefs and the percentage of farmers for which the belief is salient (n = 83).

	Normative beliefs (b) Answer scale [-2 to 2]			Motivation to comply (m) Answer scale [1 to 5]		Salient
	% NA	Mean	Stdev	Mean	Stdev	% of farmers
**Fellow farmers**	13	0.2	0.7	2.5	1.0	67
**Members of your study group**	29	0.4	1.0	2.6	1.1	54
**Your buyer**	7	0.9	0.9	3.4	1.0	87
**Farmer cooperative**	7	0.8	0.9	3.3	1.0	87
**Independent advisor**	22	0.6	0.9	2.9	0.9	70
**Government official**	39	0.2	1.1	1.9	1.0	33
**RVO**	37	0.2	1.0	2.0	1.1	34
**Fungicide supplier**	8	0.8	1.3	2.1	0.9	65
**Scientists**	20	0.7	0.9	3.0	1.1	68
**Environmental organisation**	20	0.2	1.5	1.9	0.9	45
**Consumer**	14	0.5	1.1	3.1	1.2	74
**Family members/friends**	20	0.2	0.8	2.3	1.2	51

### Correlation between beliefs and behavioural constructs

Of the five ATT beliefs, three had a positive correlation with ATT. These beliefs are ‘higher wheat quality’, ‘higher wheat yield’, and ‘lower mycotoxin contamination in wheat’ (Table 6). The belief ‘is pointless because of the unpredictability of weather conditions’ was significantly correlated to ATT when scaled according to the questionnaire answer scale, but insignificant when based on the additional four scales (Table 6). Four of the six PBC beliefs were significantly related to the PBC construct when scaled according to the answer scale (Table 7). However, when taking the other scaling options into account, only three beliefs were important because they were significantly underlying the PBC in a minimum of four out of the five scaling options. These three beliefs were: (i) ‘the availability of enough alternative preventive measures’, (ii) ‘having enough possibilities to obtain individual advice’, and (iii) ‘having sufficient materials and manpower’. All referents were significantly related to the SN when measured on the scale reflecting the answer format and when scaled on a uni-uni scale or the basic belief score. When scaled on a uni-bi scale, only four referents were significantly related to the SN, and when scaled on a bi-bi scale, only the buyer was significantly related to the SN (Table 8).

**Table 6:** Correlation coefficients between the ATT belief score (b) and the ATT construct, and between the four ATT multiplicative composites (uni-bi, uni-uni, bi-uni, bi-bi), and the ATT construct.

ATT belief	uni-bi^1^	uni-uni^2^	bi-uni	bi-bi	b
**Improves wheat quality**	0.37*	0.47*	0.47*	0.36*	0.49*
**Higher wheat yield**	0.42*	0.46*	0.44*	0.45*	0.42*
**Lower mycotoxin contamination in wheat**	0.37*	0.49*	0.52*	0.49*	0.50*
**Does not result in higher cost-effectiveness of approach**	0.07	0.10	0.08	0.04	0.10
**Is pointless because of unpredictability of weather conditions**	0.23*	0.10	-0.03	-0.01	-0.04

*p<0.05.

^1^Multiplicative composite of the belief reflecting the questionnaire answer scale: uni-bi = unipolar belief score × bipolar outcome score.

^2^Other multiplicative composites: uni-uni = unipolar belief score × unipolar outcome score; bi-uni = bipolar belief score × bipolar outcome score; bi-bi = bipolar belief score × bipolar outcome score; b = unipolar belief score.

**Table 7:** Correlation coefficients between the PBC belief score (b) and the PBC construct, and between the four PBC multiplicative composites (uni-bi, uni-uni, bi-uni, bi-bi), and the PBC construct.

PBC belief	uni-bi^1^	uni-uni^2^	bi-uni	bi-bi	b
The availability of enough alternative preventive measures	0.36*	0.52*	0.51*	0.32*	0.53*
The availability of cost-effective measures	0.20	0.36*	0.32*	0.09	0.39*
Having sufficient knowledge	0.21*	0.17	0.10	0.11	0.08
Having enough possibilities to obtain individual advice	0.34*	0.28*	0.25*	0.46*	0.14
Having sufficient materials and manpower	0.31*	0.22*	0.14	0.26*	0.08
Having sufficient financial scope to invest	0.05	0.06	0.10	0.20	0.04

*p<0.05.

^1^Multiplicative composite of the belief reflecting the questionnaire answer scale: uni-bi = unipolar belief score × bipolar outcome score.

^2^Other multiplicative composites: uni-uni = unipolar belief score × unipolar outcome score; bi-uni = bipolar belief score × bipolar outcome score; bi-bi = bipolar belief score × bipolar outcome score; b = unipolar belief score.

**Table 8:** Correlation coefficients between the SN belief score (b) and the SN construct, and between the four SN multiplicative composites (bi-uni, uni-uni, uni-bi, bi-bi), and the SN construct.

SN belief (referent)	bi-uni^1^	uni-uni^2^	uni-bi	bi-bi	b
Fellow farmers	0.37*	0.35*	0.18	-0.13	0.36*
Members of your study group	0.41*	0.35*	0.19	0.00	0.40*
Your buyer	0.42*	0.37*	0.24*	0.25*	0.40*
Farmer cooperative	0.41*	0.35*	0.21	0.21	0.41*
Independent advisor	0.44*	0.37*	0.19	0.10	0.40*
Government official	0.42*	0.49*	0.33*	-0.11	0.37*
RVO	0.33*	0.45*	0.32*	-0.15	0.33*
Fungicide supplier	0.43*	0.47*	0.28*	0.01	0.32*
Scientists	0.37*	0.35*	0.24	0.12	0.34*
Environmental organisation	0.37*	0.41*	0.19	-0.13	0.30*
Consumer	0.40*	0.34*	0.18	-0.02	0.42*
Family members/friends	0.41*	0.34*	0.14	-0.15	0.47*

*p<0.05.

^1^Multiplicative composite of the belief reflecting the questionnaire answer scale: bi-uni = bipolar belief score × bipolar outcome score (reflects the questionnaire answer scale).

^2^Other multiplicative composites: uni-uni = unipolar belief score × unipolar outcome score; uni-bi = unipolar belief score × bipolar outcome score; bi-bi = bipolar belief score × bipolar outcome score; b = unipolar belief score.

## Discussion

This study collected and analysed questionnaire data on Dutch farmers’ behaviour regarding adaptations in their future agronomic management to reduce FHB and mycotoxins in wheat, by looking into their intentions, the underlying behavioural constructs and beliefs (TPB model). To the best of our knowledge, no study investigated these aspects for agronomic management to reduce FHB and mycotoxins in wheat before. Two related studies showed that the belief ‘increase in soil fungi’ was negatively related to an attitude to increase soil organic matter in the Netherlands [35]. And the belief ‘Increase risk of fungal diseases’ was not a significant driver to adopt incorporation of crop residue in the soil (a well-known measure against Fusarium species infection), as surveyed among Italian farmers [36].

Results of this study show that 46% of the farmers had the intention to adapt their agronomic management to reduce FHB and mycotoxin contamination in wheat, whereas 21% had no such intention. No difference was found in the current agronomic management between farmers with an intention, a neutral intention, or no intention, i.e. there were no correlations between the number of measures taken and farmer’s intention (Spearman’s correlation p<0.05) and between the implementation of the benchmark approach and farmer’s intention (chi-square, p<0.05). This is in contrast to Bruijnis *et al*. [30] who found that farmers with no intention are unable to change because they already implemented multiple measures. Since there is an intention-behaviour gap, not all people with a positive intention will change their behaviour [50]. The exact percentage of farmers that will follow through with their positive intention could not be determined, because we performed a cross-sectional rather than a longitudinal study. In general, TPB models explain up to 50% of the variance for intention and less than that for predicting behaviour [51]. Hence, less than 46% of the wheat farmers with a positive intention are expected to adapt their agronomic management in the future. Reasons for not following through with the actual behaviour are a changed intention or being unable to act on the intentions because of lack of skills or environmental factors [50]. Incentive mechanisms should focus on farmers who have no intention to convert them into farmers with an intention, as well as focus on farmers who already have an intention to strengthen this intention and herewith decrease the potential intention-behaviour gap.

According to the TPB, people will have strong intentions if they evaluate the behaviour positively (ATT), if they think that there are no barriers to perform the behaviour (PBC), and if they believe that their social environment (SN) would want them to perform it [51]. The relative weights of these behavioural constructs on the intention give targets to achieve behavioural change [40]. Results show that, in general, farmers (60%) think a change in their agronomic management is beneficial (i.e. a positive ATT), an outcome that was also found by Breukers *et al*. [29]. Results of the current study showed that both ATT and SN (social environment) are positively and statistically significantly related to the intention of Dutch wheat farmers to adapt their agronomic management in the coming five years. Interestingly, PBC showed not to be a significant contributor to intention, implying that most farmers feel that they have enough opportunity to change and perceive no barriers to change. Although it was expected that PBC and related control beliefs, like financial scope and the availability of alternative pre-harvested measures, are important factors for farmers to determine to adapt their agronomic management in the future, for Dutch wheat farmers regarding *Fusarium* spp. management, this is not the case. This is in contrast to Breukers *et al*. who concluded that Dutch horticultural growers were found to be willing to apply risk management measures, and that poor risk management was mainly due to perceived barriers, such as high costs and doubts regarding efficacy of management measures [29].

To be able to stimulate Dutch farmers to adapt their agronomic management in the future, the focus should be on strengthening and changing ATT by targeting significant ATT beliefs, through the most important SN beliefs (social referents). Although the ATT belief statements studied here are specifically elicited from wheat farmers regarding agronomic *Fusarium* spp. management in the elicitation study, not all these belief statements are relevant. Only the beliefs that are related to their specific behavioural TPB construct are worth focussing on. Results show that important ATT beliefs are closely related because they focus on specific attributes of the grain, namely yield, quality, and safety (lower mycotoxin contamination) and that these beliefs are salient for most (77–87%) of the Dutch wheat farmers. If a belief is salient, it means that farmers already hold this belief and that strengthening this belief will result in a stronger behavioural construct. For the remaining farmers, the beliefs are not salient, and the focus should be on converting farmers to believe; however, the ability to change someone’s belief is a subjective judgement and cannot always be achieved in practice [50].

The standard method used for measuring beliefs in the TPB by multiplicative composites can lead to statistically uninterpretable results, the so-called ‘expectancy-value muddle’ [45–48]. The use of a single belief score might be sufficient in determining important beliefs [41]. Two studies compared this basic belief score with the multiplicative composites scores in regression models and concluded that the expectancy-value model was appropriate [52] and had only marginally better predictive power [53]. However, these studies did not test the effect of their multiplicative composites answer scales on the results, as suggested by Hardeman *et al*. [54]. In the current study, the effect of analysing the questionnaire data with different answer scales was included, to check the robustness of the results based on the standard questionnaire text scale to calculate the beliefs’ multiplicative composites. Results showed that the type of applied answer scale affected the statistical significance of the correlation between a belief and its behavioural construct (Tables 6–8). For example, in all the tested multiplicative composites, three out of five ATT beliefs were significantly correlated to the ATT construct. However, when the belief was scored on a unipolar scale and the evaluation score on a bipolar scale, an additional belief is significantly related to the ATT, suggesting that the answer scale does affect the results. This is further strengthened by the results of PBC beliefs and SN beliefs, which showed that the applied scaling influenced the outcomes and eventually the conclusions that are drawn from the study. Although the conclusions on the significant ATT beliefs are robust to the scaling used, for PBC beliefs and SN beliefs, they are not. Hence, it is important to include scaling effects to show the robustness of the results in future studies.

Interventions to strengthen or change farmers’ beliefs should go via the most important referents, namely the buyers and farmer cooperatives, because they can effectively deliver information and motivate the farmers to act. This observation means that the opinions of these social referents are important in farmers’ decision to change their approach. Consequently, if buyers and farmer cooperatives can show farmers that a change in their future agronomic management can improve their wheat quality, yield, and safety (lower mycotoxin contamination), then their belief is strengthened. Accordingly, this may lead to a definite change in agronomic management. Farmer cooperatives can achieve this, for example, by communicating these outcomes to their members through short communications, lectures, or on-farm demonstrations in which the benefits of adapting agronomic management on FHB and mycotoxin contamination are demonstrated on experimental farms.

## Conclusion

This study explored the intention, underlying behavioural constructs, and beliefs of Dutch wheat farmers to adapt their agronomic management to reduce FHB and mycotoxin contamination in wheat in the coming 5 years. Forty-six percent of the Dutch wheat farmers have the intention to adapt their agronomic management. Attitude and social norm underlie the intention of farmers to adapt their management, where as perceived behavioural control is not a significant contributor to this intention, implying farmers feel that they perceive no barriers to change. The most important underlying beliefs for attitude are ‘higher wheat yield’, ‘higher wheat quality’, ‘lower mycotoxin contamination’, and the most important referents are the buyers and farmer cooperatives. Hence, if buyers and farmer cooperatives can show farmers that a change in their future agronomic management can improve their wheat quality, yield, and safety (lower mycotoxin contamination), then a farmer’s belief is strengthened or changed, and this may lead to a definite change in future agronomic management.
